# Neuralgia Treated by Internal Use of Chloroform

**Published:** 1852-03

**Authors:** P. A. Allaire

**Affiliations:** Aurora, Ill.


ARTICEL II.

*Neuralgia Treated by the Internal Use of Chloroform.* By P. A. ALLAIRE, M. D., of Aurora, Ill.

DR. EVANS:—If the following cases of Neuralgia, treated by the internal use of Chloroform, are deemed of sufficient interest to warrant their insertion in the Journal, they are at your disposal.

*Case* 1. J. L H., aged about 40 years, of spare, but vigorous frame, of temperate and active habits, has been a resident of Northern Illinois since its first settlement. He has often suffered from the effects of malaria, but not enough to impair his general health. During the last ten years he has had many attacks of what is known as Sciatica, and for its relief has been under treatment of all kinds. During May, 1851, he first came under my care. The left limb, which had always been the seat of the disease, and was now, I found a little short, slightly everted, and its size less than the sound one; the motions are a little abridged, the effect of which is very perceptible in his gait.

The pain is dull, with occasional paroxysms of acuteness, extending over the hip and thigh, but most severe in the course of the sciatic nerve; no pain below the knee; at night the pain is usually most severe; the warmth of the bed increases it, so that often spends whole nights in an easy chair. From the character of the pain, the absence of rheumatism in other parts, and the want of heat, tenderness or swelling in the affected limb, I viewed it as a painful affection of the nerves. The present attack having lasted some three weeks, he had exhausted the remedies which he had formerly used with most benefit, and having been at different times under the whole routine of treatment and something more, without marked results, the attacks having (to use his own words) always abated when they got ready, I resolved at once to commence with the Chloroform.

May 9th. Directed eight drops to be taken on sugar at a dose, morning, noon and night, and to increase the dose two drops each day.

May 20th. Takes now twenty-five drops three times daily; feels no unpleasant effect from the Chloroform, and finds the limb decidedly more comfortable. Directed to steadily increase the dose two drops daily.

May 29th. Takes forty drops for a dose, and says he will not go any higher, as it gives him a very singular feeling of lightness, which lasts about fifteen or twenty minutes, commencing almost immediately after the medicine is swallowed. There is but little remaining uneasiness in the limb. Directed the remedy continued in 35 drop doses.

June 10th. Says he is well, and has suspended for some days the use of the medicine. The limb feels all right. Of course he continues to limp as usual, but has no pain.

Dec. 20th. Mr. H. continues in perfect health; no return of his old enemy.

*Case* 2. R. D., aged 48 years, of good general health, robust and athletic; farmer, a resident of Illinois for 8 years, and during this time has had but little malarious disease. In 1847 had neuralgia of the head and face, which was cured with Strychnine and Quinine. Since then has had good general health until May, 1851, at which time he was attacked with violent nervous pain in the back and loins. The first paroxysm lasted some four or five hours, and occurred quite regularly every week for four or five weeks, when he called on me for treatment.

June 5th. Mr. D. describes the pain as spasmodic, affecting the whole of the lower part of the back, and recurring now at irregular intervals, having no tenderness, but a feeling of weakness and numbness in the part; paroxysms vary in duration from three to six hours. His countenance has an expression of anxiety and suffering, but the various organs seem to be performing their functions in a healthy manner. The appetite is fair, except during an attack of pain.

Directed 3 grs. Mass. Hydrarg. night and morning, and 3 grs. Sulph. Quinine every two hours daily.

June 10th. Has taken the medicines as directed, and been almost daily under the influence of the Quinine, but without improvement; in fact the recurrence of pain is more frequent, and appetite not so good. Directed to increase the Quinine to 5 gr. doses, continue blue pill the same, and apply a blister 3 by 10 inches on one side of the lower half of the Spine, and when the visication had healed to apply it on the other side. Permitted to use anodynes when necessary.

June 18th. Remedies have been perseveringly used, but without benefit. Concluding that the disease was not of malarious origin, and not being able to find any structural or mechanical cause, I resolved on a thorough trial of iron. Directed the usual dose of half a drachm of the Sub Carbonate three times daily to be commenced with, and steadily increased, Morphine to be taken as occasion required, and anodyne frictions to the affected part during the paroxysms.

July 18th. Patient has lost considerable in weight—he thinks twenty pounds, looks feeble; when free from pain gets about with difficulty; at other times confined to his bed; paroxysms of pain about the same; has increased the iron to 3½ drachms at a dose; appetite capricious, digestion good, bowels regular, alvine evacuations look well; skin moist, no chills, no fever; pain excited by slight exertion, or a little mental tal excitement. Had consultation with Dr. E., and decided to continue the iron in as large doses as he could bear, anodynes the same.

July 24th. Mr. D. left home to-day for New York, and entered a water-cure establishment; no better when he started than at last report.

Oct. 14th. Mr. D. returned home. From about Aug. 15th to Oct. 1st, he has been subjected to the routine of a Hydropathic establishment, situate among the hills in Delaware Co., N. Y. Says he has gained about ten pounds in weight; that the attacks of pain are not, he thinks, quite so frequent, but are of equal severity as before.

Oct. 24th. Mr. D. requested me again to prescribe for him. Finding on careful enquiry that there was no special change in the condition of his disease, I directed the Chloroform to be tried; to begin with 5 drops three times each day, and increase the dose 2 drops daily.

Oct. 30th. Thinks he is improving; has had no paroxysmal pain of severity since last week. Takes 18 drops three times a day. Directed him to keep on increasing the dose as before, until some unpleasant effect was produced, or he recovered.

Dec. 1st. Mr. D. called to see me to-day; says he is well as ever he was; that he only took the Chloroform one week after seeing me last, and never got higher than 20 drops for a dose, as it produced a very unpleasant burning sensation in the stomach.

In concluding, permit me to make a suggestion: it is, that Chloroform may prove a useful agent in various affections, in which the nerves or their extremities may be involved, as paralysis of some kinds, epilepsy, hysteria, neuralgia, and neuralgic rheumatism. The internal use of this agent will, I believe, prove more valuable than has been supposed in nervous diseases, if we may judge from the few cases recorded; and if it should prove to be "a remedy," it will be a great boon, for their hitherto intractable nature has rendered them often a reproach to the profession.

December 20th, 1851.

